# Sequence-based Association Analysis Reveals an *MGST1* eQTL with Pleiotropic Effects on Bovine Milk Composition

**DOI:** 10.1038/srep25376

**Published:** 2016-05-05

**Authors:** Mathew D. Littlejohn, Kathryn Tiplady, Tania A. Fink, Klaus Lehnert, Thomas Lopdell, Thomas Johnson, Christine Couldrey, Mike Keehan, Richard G. Sherlock, Chad Harland, Andrew Scott, Russell G. Snell, Stephen R. Davis, Richard J. Spelman

**Affiliations:** 1 Livestock Improvement Corporation, Hamilton, New Zealand; 2 School of Biological Sciences, University of Auckland, Auckland, New Zealand

## Abstract

The mammary gland is a prolific lipogenic organ, synthesising copious amounts of triglycerides for secretion into milk. The fat content of milk varies widely both between and within species, and recent independent genome-wide association studies have highlighted a milk fat percentage quantitative trait locus (QTL) of large effect on bovine chromosome 5. Although both *EPS8* and *MGST1* have been proposed to underlie these signals, the causative status of these genes has not been functionally confirmed. To investigate this QTL in detail, we report genome sequence-based imputation and association mapping in a population of 64,244 taurine cattle. This analysis reveals a cluster of 17 non-coding variants spanning *MGST1* that are highly associated with milk fat percentage, and a range of other milk composition traits. Further, we exploit a high-depth mammary RNA sequence dataset to conduct expression QTL (eQTL) mapping in 375 lactating cows, revealing a strong *MGST1* eQTL underpinning these effects. These data demonstrate the utility of DNA and RNA sequence-based association mapping, and implicate *MGST1*, a gene with no obvious mechanistic relationship to milk composition regulation, as causally involved in these processes.

Milk lipids comprise the predominant energy source of the developing neonate in most mammalian species. There is substantial inter- and intra-species variation in milk fat content^1^, and in cattle populations, the genetic architecture regulating milk fat percentage appears to consist of many quantitative trait loci (QTL) contributing small effect, with a handful of loci explaining relatively large proportions of the genetic and phenotypic variance^2^,^3^. Several of the causative genes underlying these larger QTL have now been confirmed, including *DGAT1*^4^, *ABCG2*^5^, *GHR*^6^, and *AGPAT6*^7^. The existence of a major QTL affecting milk fat percentage on bovine chromosome 5 has also been reported in numerous independent studies^3^,^7^,^8^,^9^,^10^,^11^. Although *EPS8* and *MGST1* have been speculated to underlie these effects, the causative status of these genes has not been functionally demonstrated.

Given the economic importance of bovine dairy products and beef as human food sources, considerable genetic resources exist for the study of complex traits in *Bos taurus*. This includes SNP-array panels used for genome-wide association studies, and like the evolution of human genotyping platforms, the density of these panels has steadily increased over time. Driving this increase in SNP-density is the underlying principal that for association mapping to be effective, SNPs must be in sufficient linkage disequilibrium with causal variants to discern genetic signal^12^. In this regard, using genome sequence-derived variant panels provides the ultimate resolution, since the association between causative variants and the trait can be tested directly. The cost to conduct whole genome sequencing in very large populations is still prohibitively high, however, integrating whole genome sequence reference panels with SNP-array data may allow imputation of sequence-resolution genotypes^13^,^14^. So far, the number of studies demonstrating the use of this strategy has been limited, though recent examples include the discovery of putative causative variants in cattle^15^,^16^,^17^, and human disease^18^,^19^.

In the current study, we performed a detailed investigation of a major QTL affecting bovine milk fat percentage. Bayesian genome-wide association analysis was conducted in a large population of outbred cows to first estimate the genomic location of the QTL. A reference panel of 556 whole genome sequences was then used to impute sequence-resolution genotype data at the chromosome 5 locus, with association analysis revealing a handful of candidate causal variants for this QTL, and a variety of other milk components. We then utilise high depth mammary RNA-sequence data to examine the expression of candidate causative genes for these effects, identifying a large expression QTL (eQTL) for a single gene that is not only highly correlated with the milk composition QTL, but harbours the top associated variants within its proximal regions.

## Results and Discussion

### Genome-wide association analysis identifies a chromosome 5 QTL for milk fat percentage in a large population of outbred dairy cattle

**Figure 1:**
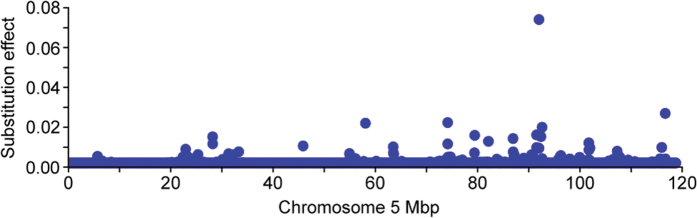
Milk fat percentage QTL on chromosome 5. Chromosome 5 Manhattan plot showing the milk fat percentage QTL at 93.9 Mbp representing 42,933 cows. The X-axis shows Mbp position on chromosome 5 according to the UMD3.1 *Bos taurus* reference assembly, the Y-axis shows the absolute value of the allele substitution effect on milk fat percentage as estimated using a Bayes B model.

Bayes B association mapping was conducted using 437,255 SNPs imputed from the Illumina BovineHD BeadChip in an outbred cow population of 42,933 New Zealand Holstein-Friesians (HF), Jerseys (J), and HFxJ crossbreeds. The percentage of phenotypic variance explained by all fitted SNPs in the Bayes B model was 35.6%. Consistent with previous genome-wide association studies (GWAS) of milk fat percentage^3^,^7^,^8^,^9^,^10^,^11^, a major QTL was detected on chromosome 5, with the greatest effect estimated for the BovineHD0500026662 SNP (g.93945655G > T; Fig. 1; Supplementary Table S1 and S2). Notably, this was the same lead SNP reported by the subset of previous studies that also used the Illumina BovineHD platform^7^,^9^,^10^. Using empirical gene structure information derived from mammary RNA sequence (RNA-seq) data, this SNP mapped to intron 1 of *MGST1*, ~186 kbp downstream of the *LMO3* gene, and ~364 kbp upstream of the *DERA* gene. When the genome was partitioned into 1 Mbp windows and the combined effects of SNPs within each interval estimated, the chromosome 5 93-94 Mbp window accounted for 2.1% of the genetic variance in milk fat percentage, representing the third largest QTL genome-wide (Supplementary Table S2). Of the top five genome-wide window QTL, all except the chromosome 5 93-94 Mbp locus contained genes with previously demonstrated causal roles in milk composition regulation. These were: chromosome 14 1-2 Mbp (*DGAT1*^4^), chromosome 27 36-37 Mbp (*AGPAT6*^7^), chromosome 11 103-104 Mbp (*PAEP*^20^), and chromosome 20 31-32 Mbp (*GHR*^6^).

### Chromosome 5 sequence imputation and accuracy assessment

Having estimated the location of the chromosome 5 QTL, we then imputed sequence-based genotype data into a 10 Mbp interval centred on the BovineHD0500026662 SNP for a population of 64,244 animals. This population comprised the 42,933 animals used for the original GWAS, and an additional 21,311 animals (of similar breed composition) typed predominantly using the Super GGP BeadChip. The sequence reference panel consisted of 556 animals at an average of 15× genome-wide mapped read depth. These animals were broadly representative of the New Zealand dairy population, where 18,854 of the 64,244 animals targeted for imputation had a sire directly represented in the sequence reference, and an additional 28,564 animals had at least one grandsire represented. Variants were called from sequence alignments using RTG Core^21^, yielding 109,517 variants in the 10 Mbp target interval. These variants were then imputed into the phenotypic population using Beagle software (v4)^22^, applying a stepwise imputation strategy to integrate data across different genotyping platforms. The imputation strategy and animal populations are described in greater detail in Materials and Methods.

**Figure 2:**
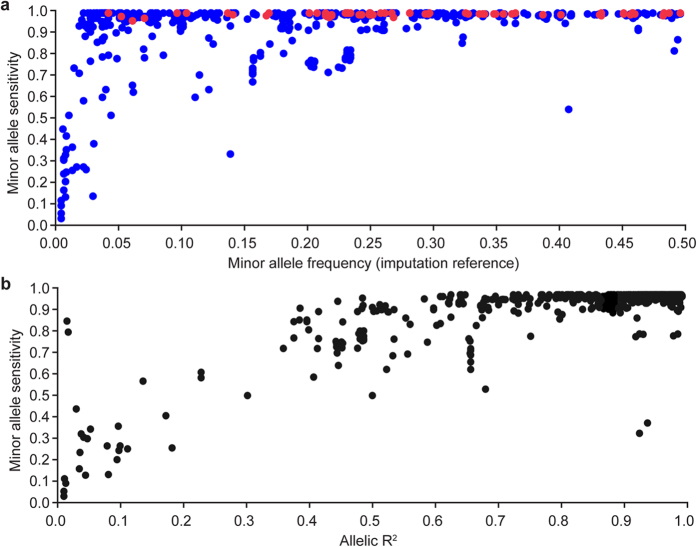
Accuracy of genome sequence-resolution imputation. Scatter plots showing imputation accuracy (minor allele sensitivity) of imputed sequence variants relative to minor allele frequency (2A), and allelic R^2^ (2B) values. These comparisons used 790 SNPs common to both imputed genome and RNA-seq-derived genotype sets, where variants called from the RNA-seq alignments were used to assess sequence imputation accuracy in these 406 animals. Red variants shown in panel 2A represent the subset of real (i.e. not imputed) genotypes in the genome sequence-derived dataset (70 SNPs from the Illumina BovineHD chip), giving an indication of the accuracy of variant calls derived from the RNA-seq-alignments.

Four hundred and six animals targeted for imputation were also subjected to mammary RNA-seq. To estimate the accuracy of imputation, imputed genome sequence-derived genotypes were compared to SNPs called directly from the RNA-seq alignments. These data were considered an independent dataset, since only SNP-chip genotypes were used for sequence imputation in these animals. RNA-seq derived genotypes were first filtered to provide a robust call set of ‘real’ genotypes suitable for concordance calculations (Materials and Methods). For the subset of 994 filtered SNPs that mapped to the 10 Mbp imputation interval, 790 were common to the genome sequence-derived dataset. For these 790 SNPs, the overall accuracy of imputation was high (median minor allele sensitivity (MAS) = 0.99; mean MAS = 0.93; Supplementary Table S3). As expected, more frequent variants were more accurately imputed (Fig. 2A), and as reported by others^22^,^23^, the Beagle allelic R^2^ metric was a robust indicator of imputation accuracy (where median MAS = 0.99, mean MAS = 0.98 for variants with allelic R^2^ > 0.7; Fig. 2B). Notably, the accuracy of imputation for variants with MAF > 0.20 was also very high (median MAS = 0.99; mean MAS = 0.98), of key relevance given the allele frequency of the lead tag-SNP from milk fat percentage association analysis (BovineHD0500026662 MAF = 0.27 in the reference population, MAF = 0.41 in the RNA-seq cohort). Assuming that the BovineHD0500026662 GWAS tag-SNP was in moderate LD with the underlying QTL, these data suggested that the vast majority of candidate causative variants would be accurately imputed in this sample.

### Sequence-based association analysis highlights a cluster of candidate causal variants mapping to 5′ proximal regions of the *MGST1* gene

For association testing, analysis was restricted to a 2 Mbp target interval centred on the top chromosome 5 tag-SNP from GWAS (BovineHD0500026662; chr5:92945655-94945655). Filtering to remove multi-allelic and low frequency variants (MAF < 0.005) yielded 13,719 SNPs, 863 indels, 255 multiple nucleotide polymorphisms (MNPs), and 107 ‘mixed’ variants (class combinations) for analysis. Of these 14,944 variants, 14,448 were derived wholly from genome sequence data, with 496 (3.3%) present on one or more of the Illumina BovineHD, BovineSNP50, or Super GGP BeadChip platforms used for physical genotyping. Quantifying imputation accuracy as described above, coincident RNA-seq variants in this 2 Mbp interval had a median MAS = 0.99, and mean MAS = 0.97 (N = 25 SNP; Supplementary Table 3).

**Figure 3:**
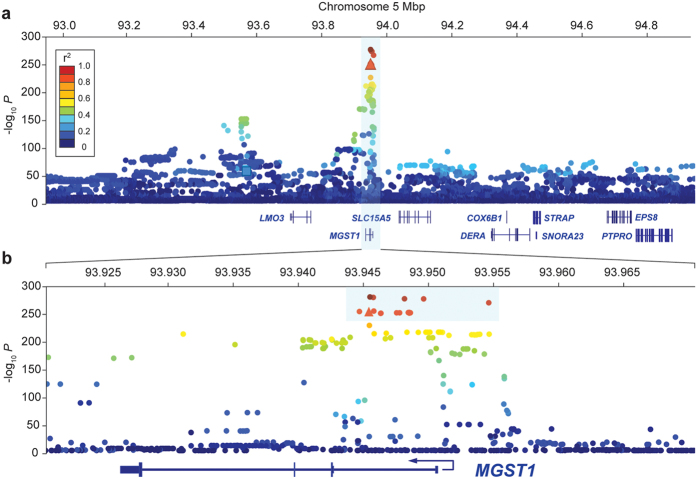
Association mapping of milk fat percentage. Manhattan plots showing association of sequence variants with milk fat percentage in an outbred cow population of 64,139 animals. Panel 3A shows a 2 Mbp interval of variants (chr5:92945655-94945655) centred on the SNP of greatest effect from Bayes B GWAS (BovineHD0500026662), panel 3B shows a zoomed, 50 kbp view of the locus showing peak association (chr5:93920738-93970738). The nine genes annotated to the 2 Mbp locus are indicated, with the zoomed panel showing the distribution of associated variants across the *MGST1* gene structure. Variants are coloured relative to their linkage disequilibrium (R^2^) relationships with the top variant from sequence-based association (g.93945738C > T), with variants highlighted by the blue box (3B) comprising the cluster of 17 highly associated, highly correlated SNPs considered as potential functional candidates for the QTL. The top-associated Illumina BovineHD and SNP50 panel SNPs are indicated by the triangle (BovineHD0500026662), and square (Hapmap36414-SCAFFOLD150043_20489) respectively.

Associations between sequence-variants and milk fat percentage were assessed using pedigree-based mixed models in ASReml-R^24^. Peak significance was observed for a genome sequence-derived SNP g.93945738C > T (*P* = 1.98 × 10^−292^; Fig. 3A,B, Supplementary Table S4), comprising one of a cluster of 17 SNPs in strong linkage disequilibrium (LD) (R^2^ > 0.80). Although *P*-values for this cluster of variants spanned many orders of magnitude (Table 1), we considered any one of these as potentially causal, since errors in genotyping, imputation, and phenotyping could potentially obscure the true variant association rankings^25^. This cluster included the Illumina BovineHD SNP-chip variant BovineHD0500026662 (*P* = 8.89 × 10^−265^; R^2^ = 0.83 with g.93945738C > T), though notably, tagging of the QTL signal was relatively poor for BovineSNP50 chip variants (Fig. 3A; smallest *P* = 5.77 × 10^−60^ for Hapmap36414-SCAFFOLD150043_20489 g.93567346G > A; R^2^ = 0.27 with g.93945738C > T). Based on the *MGST1* gene structure derived from mammary RNA-seq data, the cluster of 17 top-associated SNPs were all non-coding variants spanning a 10 kbp interval from intron 1 to 4 kbp upstream of the transcription start site (TSS; Fig. 3B). The g.93945738C > T SNP explained 4.6% of the genetic variance and 2.8% of the phenotypic variance in milk fat percentage in the population of 64,139 animals. Relative to g.93945738 ‘TT’ individuals, this translated to a 2.3% increase in milk fat percentage per ‘C’ allele.

Fitting the g.93945738 genotype as a covariate in the association models and rerunning the analysis for the remaining variants removed most of the association signal in the 2 Mbp interval (Supplementary Table S5 & Supplementary Figure S6). Although some residual signal remained, this appeared to be dispersed over the broader locus, and may have represented population stratification incompletely accounted for in the pedigree-based models, and/or valid association signal from other genes. Wang *et al.*^3^ reported mapping of a milk fat percentage QTL of large effect in German Holstein-Friesian cattle using the Illumina BovineSNP50 chip, locating to a similar position to that reported here. The authors attributed this signal to *EPS8* as a candidate gene, and based on transcription factor binding site prediction, proposed a putative promoter SNP (g.94553580T > C) as a potential causal mutation for this effect^3^. Raven *et al.*^9^ also reported highly significant effects for this locus using the Illumina BovineHD chip. Peak significance was observed for the same intronic *MSGT1* variant identified from Bayesian GWAS in the current study (BovineHD0500026662), though the authors also acknowledged a potentially independent signal located nearer *EPS8*^9^. We did not observe an association peak overlapping *EPS8* in our dataset, and notably, the candidate mutation g.94553580T > C proposed to underlie this QTL is polymorphic in our sample (MAF of 0.134 in our genome sequence reference). Imputation of the variant yielded an allelic R^2^ of 0.88, suggesting the variant was imputed accurately in our population (Fig. 2B; Supplementary Table S4), and although the SNP was significantly associated with milk fat percentage in our primary analysis (*P* = 3.26 × 10^−44^), fitting the lead g.93945738 *MGST1* genotype removed this effect (*P* = 0.561). These data do not preclude the possibility that another downstream, non-segregating QTL exists, but suggest the *EPS8 *g.94553580T > C variant would be unlikely to underlie such an effect.

### Pleiotropy of other milk components

**Figure 4:**
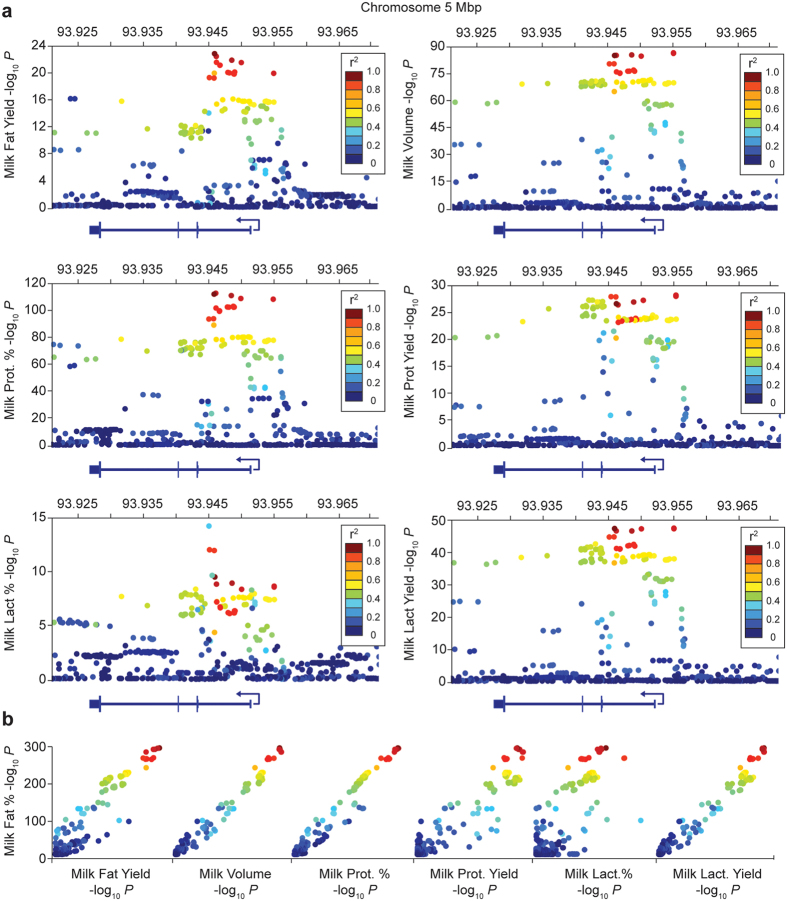
Milk composition and yield effects at the chromosome 5 locus. Manhattan plots (4A) showing association of sequence variants for multiple milk composition and yield traits at the *MGST1* locus (chr5:93920738-93970738; *MGST1* gene structure is indicated in each panel). The number of animals included in these analyses range from 64,141 to 51,120 between traits (Table 2). Figure 4B shows scatter plots of –log_10_ *P*-values where the y-axis represents the milk fat percentage association statistic and the x-axes represent association for the other milk traits. These plots indicate the rank order of variants relative to milk fat percentage, with the strong correlation of signals suggesting a common genetic basis for all traits. Variants are coloured according to their linkage disequilibrium (LD) with the top milk fat percentage associated SNP g.93945738C > T, with LD reflecting values calculated within each respective population.

We have previously noted pleiotropic effects for milk traits in cattle, whereby genetic variants co-ordinately impact a diverse range of milk components (e.g. variants in the *DGAT1* and *AGPAT6* fat synthesis enzymes^4^,^7^). To look for similar effects, we conducted association analysis of milk protein, fat, and lactose yield, milk volume, and milk protein and lactose percentage in the large outbred population. Using a refined, 50 kbp interval of sequence-based variants centred on the g.93945738C > T *MGST1* SNP (632 variants), significant associations were demonstrated for all milk traits tested (Fig. 4A, Table 2, Supplementary Table S7). In the case of milk protein percentage, milk volume, and lactose yield, these effects were substantial, with the g.93945738C > T *MGST1* SNP explaining ~2% or more of the genetic variance for each trait (Table 2). The high milk fat percentage g.93945738 ‘C’ allele was associated with increased fat yield, protein percentage and lactose percentage, whereas decreases were observed in protein yield, lactose yield, and milk volume. These effects are consistent with the findings of Kemper *et al.*^10^, where impacts on a subset of the same milk composition traits (of corresponding sign) were also observed at this locus.

All milk composition effects appeared to derive from the same underlying genetic signal, with the same cluster of 17 high-ranking milk fat percentage-associated variants also ranking highly among the other milk traits. To quantitatively assess these relationships, we performed Spearman’s rank correlation analysis between the *P*-value results lists for these traits and milk fat percentage. This analysis revealed strong correlations between QTLs (Fig. 4B), particularly between milk fat and protein percentage (ρ = 0.92), where these two phenotypes also corresponded to the two most significant QTLs among all traits. These data suggested a common genetic signal driving multifaceted changes in milk composition, and further pointed to one of the 17 non-coding *MGST1* candidate SNPs as likely underpinning these effects.

### *MGST1* expression associates with milk fat percentage QTL genotype in lactating mammary tissue

**Figure 5:**
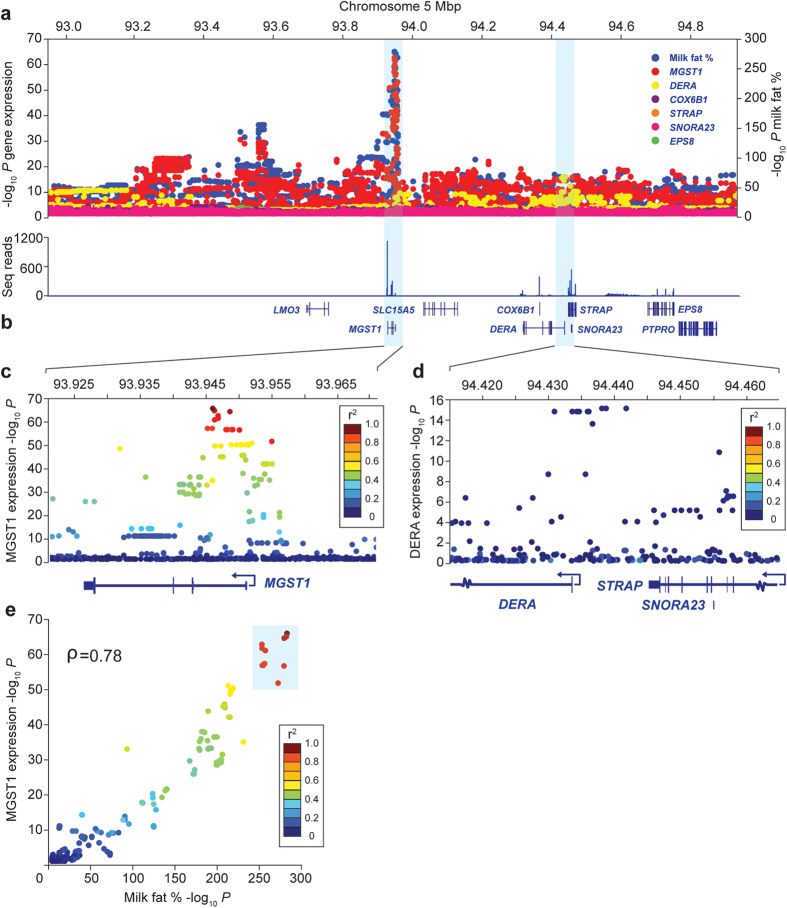
Expression analysis of the chromosome 5 milk composition locus. Figure 5A shows the same 2 Mbp milk fat percentage association results represented in Fig. 3A, overlaid with additional eQTL plots for the six mammary-expressed genes at this locus. Association results for these six genes share the same y-axis (left) and are differentiated by colour. Figure 5B shows an RNA-seq expression histogram of the locus, indicating mean read counts for these six genes expressed as an average over all 406 sequenced individuals. Figures 5C,D show 50 kbp exploded views of the two statistically significant eQTL, (*MGST1* – red, and *DERA* - yellow), with variants coloured according to their linkage disequilibrium with the top milk fat percentage-associated SNP g.93945738C > T. Figure 5E shows the rank order of associated variants between the milk fat percentage QTL and *MGST1* eQTL, plotting –log_10_ P-values for each phenotype on the x and y axes respectively. Both QTLs share the same lead variant (g.93945738C > T), part of the cluster of 17 top-ranked variants identified in analysis of milk fat percentage (demarcated by the blue box in Figure 5B,E).

The absence of protein-coding polymorphisms from the list of top trait-associated variants suggested an expression-based mechanism underlying the milk composition QTLs. To look for underlying expression QTLs (eQTL), we used high-depth mammary RNA-seq data representing the same 406 lactating cows used to quantify imputation accuracy. Of the nine annotated genes in the 2 Mbp interval of interest, six were appreciably expressed (*MGST1*, *DERA*, *COX6B1*, *STRAP*, *SNORA23,* and *EPS8)*, with *MGST1, COX6B1,* and *SNORA23* abundantly expressed (>20 fragments per kilobase of exon model per million mapped (FPKM); Fig. 5B). Expression QTL mapping was conducted using normalised read counts representing these six genes in a quality-filtered subset of 375 animals (Materials and Methods). These models tested 14,971 imputed sequence variants, representing slightly more variants than the set used for milk composition analysis (due to differential impacts of MAF filtering between populations). Association analysis revealed highly significant *cis* eQTL for the *MGST1* and *DERA* genes (*P* = 3.16 × 10^−66^ and *P* = 3.31 × 10^−16^ respectively; Fig. 5A,C,D; Supplementary Table S8). Notably, no effect on expression was apparent for *EPS8*, with the proposed *EPS8* regulatory variant^3^ g.94553580T > C un-associated with expression (*P* = 0.22; Supplementary Table S8). The *MGST1* and *DERA* eQTLs explained large proportions of the phenotypic and genetic variance in gene expression, with the top associated *MGST1* variant explaining 60.7% and 88.8% respectively, and the top *DERA* variant explaining 18.8% and 59.1% respectively. Peak signal for *DERA* expression was 500 kbp downstream of the milk composition signals, deriving from a cluster of 10 variants spanning the first intron, 5′UTR, and non-coding sequence immediately upstream of the *DERA* TSS (Fig. 5D). Critically, the *MGST1* eQTL located to the same position as the milk composition QTLs, and shared the same lead SNP (g.93945738C > T) with the milk fat percentage analysis (Fig. 5E). Manual inspection of sequence alignments in animals of opposing eQTL genotype revealed uniform transcript structures with splice sites conforming to the Ensembl reference annotation, suggesting a transcriptional (versus splicing) based mechanism to the eQTL. As with correlation analyses between the different milk composition QTLs, *P*-values for the *MGST1* expression and milk fat and protein percentage phenotypes were highly correlated (ρ = 0.73; Fig. 5E), where the g.93945738 ‘C’ allele corresponded to increased *MGST1* expression and increased milk fat percentage. These data suggested that variants affecting expression of *MGST1* were responsible for the differences in milk composition demonstrated at this locus, confirming this as the causative gene for these effects.

### Trans eQTL analysis of milk composition QTL genotype shows limited co-associated gene expression networks

Unlike the *DGAT1* and *AGPAT6* fatty-acid synthesis enzymes that underlie other major milk production QTLs^4^,^7^, the mechanistic explanation for the impact of *MGST1* on these traits is unclear. The *MGST1* gene encodes a glutathione S-transferase that protects against oxidative stress, responsible for conjugation of glutathione to reactive intermediates. The enzyme is localised to the mitochondrial membrane and endoplasmic reticulum, and known functions include aspects of lipid biology, where *MGST1* has been shown to reduce lipid peroxidation products in human mammary cell culture^26^. Whether these protective roles could quantitatively impact milk synthesis and secretion, either through reduction of oxidised forms of fatty acids, or protection of the secretory cells and their machinery, is unknown. Equally plausible roles might involve signalling functions akin to the roles of other glutathione S-transferase family members^27^, or mitochondrial-based functions relating to the increased metabolic demands of lactation. To attempt to provide insight into these mechanisms, we conducted a *trans* eQTL analysis by looking for genes co-expressed by milk composition/*MGST1* eQTL genotype. This analysis used ASReml-R to fit similar models to those used for SNP association mapping, in this case testing the effect of the g.93945738C > T genotype on the 9,348 nominally expressed genes in our sample of 375 lactating cows. After accounting for multiple testing, this analysis did not reveal any additional, significantly differentially expressed genes (Supplementary Table S9). These results suggest that we were either underpowered to detect such effects, or that *MGST1* is operating at the terminal end of the relevant expression networks. If the increases in fat yield and percentage elicited through increases in *MGST1* expression indeed derive from its enzymatic role as a detoxification enzyme, then this lack of concomitant expression might be anticipated.

### Reference assembly refinement reveals additional variants at the chromosome 5 milk fat percentage locus

**Figure 6:**
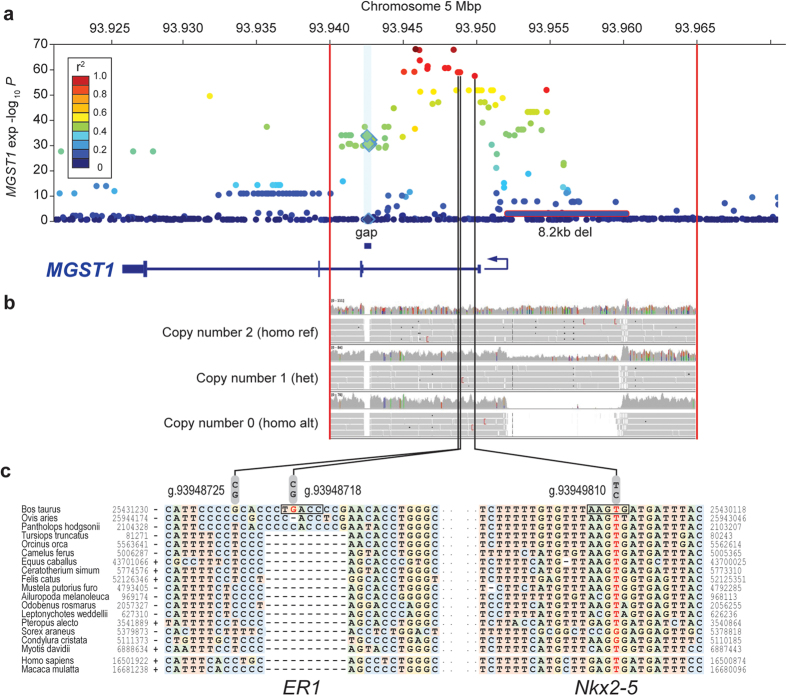
Variant curation and transcription factor binding site analysis. *MGST1* gene expression Manhattan plot (6A) using a re-imputed 2 Mbp interval of sequence variants following reference assembly refinement and discovery of additional variants at the chromosome 5 milk composition locus. Gap variants are indicated by diamonds and coloured according to their linkage disequilibrium with the lead SNP (g.93945738C > T). The 8.2 kbp deletion variant is indicated, with its association representing haplotype-based results. Representative genome sequence alignments showing genotype classes of the biallelic deletion are also shown (6B). Figure 6C shows the evolutionary conservation of sequence flanking the g.93948718G > C and g.93949810G > A SNPs with predicted impacts on putative transcription factor binding sites. Bovine sequences within these alignments represent the minus strand and thus are reverse orientation to that depicted in Fig. 6A.

We noted that the cluster of 17 highly associated MGST1 variants were adjacent to a reference sequence gap present in both UMD3.1 and Btau_4.6.1 assemblies. To identify potentially functional variants located within the 364 bp gap (UMD3.1), we used a sequential re-mapping, gap extension, and *de novo* assembly strategy to reveal an additional 17 variants in the 556 reference animals (Materials and Methods). Following the same imputation and association analysis pipeline used previously, none of the gap variants approached the significance of the original top 17 variants (milk fat percentage smallest *P* = 1.12 × 10^−214^ c.f. *P* = 1.62 × 10^−292^; *MGST1* expression smallest *P* = 3.97 × 10^−40^ c.f. *P* = 3.6 × 10^−66^; Fig. 6A; Supplementary Table S10). This suggested these previously hidden variants were unlikely to be the source of the milk fat percentage QTL signal.

Through manual inspection of sequence alignments, we also noted read depth anomalies upstream of the *MGST1* TSS, and analysis using CNVnator^28^ software indicated the presence of a large, polymorphic deletion (Fig. 6B). Subsequent PCR amplification and sequencing of the putative breakpoints confirmed the presence of an 8.2 kbp BovB repeat-flanked deletion in a proportion of individuals (Materials & Methods). As an obvious candidate mutation for the milk composition and expression QTLs, we next used the genotyping function of CNVnator to call genotypes on the manually refined CNV interval. This analysis suggested a biallelic mutation with a MAF of 0.15, and identification of a perfectly correlated BovineHD SNP-chip-derived haplotype encompassing the deletion (R^2^ = 1) suggested accurate sequence-based calling of the variant (Materials and Methods). To assess phenotypic consequences of the variant, we conducted association analysis of *MGST1* gene expression using this proxy-haplotype, targeting the RNA-seq cohort since 95% of these animals had been physically genotyped using the BovineHD chip. Using a haplotype dosage model identical to those used for SNP-based eQTL mapping, this analysis did not support the deletion as the likely causative mutation for *MGST1* expression (*P* = 0.006 c.f. smallest *P* = 3.16 × 10^−66^; Fig. 6A). Notably, two of the original 17 highly associated SNPs (g.93954748 & g.93954751) collocated to the deleted segment. Raven *et al.*^15^ also highlighted one of these variants (g.93954751) as the top milk fat percentage-associated SNP at the locus in a similar analysis incorporating imputed genome sequence data. Although it is unknown whether the 8.2 kbp deletion segregates in both populations, haplotype analysis removed these variants as plausible functional candidates in our study given that a). they were located within the un-associated CNV, and b). the confounding effect of hemizygous and homozygous deletion states on these variants would render their imputed genotypes incorrect. Although it is possible that additional variants that were difficult to sequence, call, or impute were overlooked at this locus, overall these data pointed back to 15 of the original 17 highly associated SNPs as the most likely functional candidates.

### Bioinformatic analyses of candidate causal variants

To investigate the effect of the 15 top-ranked SNP variants on gain or loss of potential transcription factor binding sites, we scored the reference and variant sequences using Matrix Search for Transcription Factor Binding Sites (MATCH)^29^ in conjunction with the TRANSFAC (Release 7.0) database. Using a combination of MATCH thresholds (Materials and Methods), the g.93948718G > C and g.93949810G > A SNPs were predicted to impact putative binding sites (Fig. 6C). The g.93949810G allele was predicted to result in loss of a binding site for Nkx2-5, however, *NKX2-5* transcripts were not detected in our mammary gland dataset, suggesting that this is unlikely to underlie the milk composition and expression QTLs. The predicted loss of an ESR1 binding site for the g.93948718G allele is of note, as this SNP is located in a 10 bp sequence unique to cattle, sheep, and Tibetan antelope (Fig. 6C), but is absent in all other mammalian genome assemblies. The ESR1 transcription factor is a nuclear oestrogen receptor critical to mammary development and differentiation^30^, and although its relevance following the onset of milk production is not well documented, *ESR1* expression increases as the course of lactation progresses^31^. Although functional testing will be essential to pinpointing the individual element underlying these QTLs, these data suggest a plausible candidate mutation and transcription factor for future mechanistic investigation.

## Conclusions

We present a detailed analysis of a pleiotropic milk composition QTL on bovine chromosome 5, demonstrating large effects for milk fat and protein percentage, milk volume, and milk lactose yield. Integrating large scale DNA and RNA sequence datasets, we provide strong evidence for the *MGST1* gene underpinning these QTLs, highlighting a major *MGST1 cis* eQTL that shares the same, highly associated sequence variants implicated in analyses of milk composition. We report detailed sequence curation of the *MGST1* locus, uncovering a number of previously obscured variants including an 8.2 kbp deletion variant 5′ of the *MGST1* TSS. Despite the outstanding biological candidacy of this variant, this deletion appears unlikely to underlie the gene expression and milk composition QTLs, though we identify alternative candidate causative polymorphisms as a basis for future functional investigation. Most remarkably, confirmation of *MGST1* as the causal gene impacting this diverse array of milk composition phenotypes offers little clue as to the mechanism of effect. Although MGST1 is known to directly bind fatty acids, these activities appear to relate to its role as a detoxification enzyme, so the potential impact on milk lipid synthesis and/or secretion is unknown. As such, elucidation of this mechanism may provide insight into new mechanisms of lactation biology, and at a practical level, future work to tease apart the molecular contribution of the candidate variants should provide opportunities for animal selection and/or gene editing.

## Materials and Methods

### Ethics statement

All animal experiments were conducted in strict accordance with the rules and guidelines outlined in the New Zealand Animal Welfare Act 1999. Most data were generated as part of routine commercial activities outside the scope of that requiring formal committee assessment and ethical approval (as defined by the above guidelines). For the mammary tissue biopsy experiment, samples were obtained in accordance with protocols approved by the Ruakura Animal Ethics Committee, Hamilton, New Zealand (approval AEC 12845). No animals were sacrificed for this study.

### Availability of materials and data

Primary datasets consisting of relevant genotypes, phenotypes, and sequence data representing all reported populations have been deposited into the Dryad digital data repository^30^, and NCBI Short Read Archive (SRP067001).

### Animal populations and milk composition analysis

The animal populations used for milk production association analysis consisted of 64,142 female dairy cows located on commercial dairy farms throughout New Zealand, forming part of a large phenotypic and genotypic database of animals used for commercial evaluation of sire performance. Differences in the number of animals quoted for analysis of different traits reflect impacts of genotype and phenotype quality filtering criteria applied for the different traits. All animals were born between 1998 and 2011, with > 95% of records derived from animals born after 2004. When ‘purebred’ animals were defined as having a breed proportion of at least 14/16ths, this population consisted of 20,958 Holstein-Friesians, 11,704 Jerseys, 30,979 Holstein-Friesian x Jersey crossbreeds, and 501 animals that were more than 4/16ths other breeds. Other breed proportions that were also present in these animals included Ayrshire, Brown Swiss, Guernsey, Hereford, Milking Shorthorn, and Swedish Red. The concentrations of major milk components were measured as part of standard herd-testing procedures using Fourier transform infrared spectroscopy. Most milk samples were processed by LIC Testlink (Newstead, Hamilton, New Zealand) using the MilkoScan FT6000 instrument (FOSS, Hillerød, Denmark). For association analyses, milk composition records were restricted to first lactation measurements from October to January inclusive. The 406 animals used for RNA-sequencing consisted of 215 animals described in a previous study^7^, and an additional 191 samples of mostly Holstein Friesian ancestry.

### DNA Extraction and SNP-chip Genotyping

Genomic DNA extraction for high-throughput genotyping was conducted using ear-punch tissue samples or blood, processed using Qiagen BioSprint kits (Qiagen) or a MagMAX system (Life Technologies). The former extraction method was performed by GeneMark (Hamilton, New Zealand), with the latter performed by GeneSeek (Lincoln, NE, USA). For whole genome sequencing, DNA was extracted using phenol-chloroform-based protocols, using semen samples from males, and blood or ear punch tissue samples from females. All high throughput genotyping was conducted by GeneSeek (Lincoln, NE, USA), with animals typed across a range of platforms including the GeneSeek Genomic Profiler BeadChip (Super GGP; GeneSeek/Illumina; N = 26,878), the Illumina BovineSNP50 BeadChip (Illumina; N = 36,266), or the Illumina BovineHD BeadChip (Illumina; N = 1,038). Most (N = 377) of the 406 RNA-seq animals were typed using the BovineHD BeadChip, with the remainder (N = 29) typed using the BovineSNP50 platform.

### Whole genome sequencing

Whole genome sequencing was conducted on 556 individuals, consisting of 285 animals from an experimental crossbreed pedigree^7^,^31^,^32^, and a collection of 271 outbred sires and dams representing the wider commercial dairy population of New Zealand. Outbred individuals were selected for sequencing based on specific phenotypes of interest, or their representativeness of the population structure found in New Zealand. Using the same breed criteria outlined above, the sequenced animals consisted of 137 Holstein-Friesians, 100 Jerseys, 318 Holstein-Friesian x Jersey crossbreeds, and 1 Ayrshire. Whole genome sequencing has been described previously^33^; briefly, 100-bp paired-end sequencing was performed by Illumina FastTrack using the Illumina HiSeq 2000 instrument. Sequencing was conducted in several phases with libraries prepared and sequenced using Illumina PCR-based protocols and v2 chemistry (N = 25 samples), and PCR-free libraries in conjunction with v3 chemistry (N = 531 samples). A range of read depths were targeted resulting in a total mapped yield of >8 tera-bases (see ‘Genome sequence informatics’ section below).

### Genome sequence informatics

#### Read-mapping and variant calling

Paired-end FASTQ data were aligned to the *Bos taurus* UMD3.1 genome assembly using BWA MEM 0.7.8^34^, with duplicates marked using Samtools rmdup 0.2.0-rc11^21^. This yielded an average of 15× mapped read depth with a median of 8× for the 556 samples (with depth ranging from 5× to 161×). RTG Core 3.3.2^35^ was then used to call variants for the chromosome 5 10 Mbp interval of interest (chr5:88945655-98945655), yielding 109,517 variants (using default filtering criteria).

#### Locus-specific assembly refinement

To determine the sequence of the 364 bp reference gap in *MGST1* intron 1, reads representing this gap were identified by a sequential re-mapping and gap extension strategy in two unrelated animals. *De novo* assembly of the reads resulted in a non-reference consensus sequence of 410 bp, which was confirmed by PCR and Sanger sequencing (see ‘PCR and targeted sequencing’ section below). A modified reference sequence was then derived by replacing the 346 gap N’s with the consensus sequence. For each of the 556 genome-sequenced animals, all reads that were unmapped, not mapped as proper pairs, or were properly mapped to a 1 Mbp interval spanning the gap (chr5:93430000-94430000), were then re-mapped to the gap-filled reference using BWA^36^, with duplicates marked (Picard MarkDuplicates v1.89^37^) and indel realignment performed (GATK indel realigner 3.1.7;^38^). RTG Core was then used to identify 17 new variants in this gap for the 556 animals, using identical variant calling criteria to that applied for analysis of whole genome assemblies.

### Sequence imputation

Sequence-based genotypes representing the 556 reference animals were imputed into the phenotypic population of 64,244 cows (animals with milk composition phenotypes and/or RNA-sequence data) using Beagle v4^22^. Imputation was conducted in a step-wise manner, where target animals with Super GGP and BovineSNP50 genotypes were first imputed to the BovineHD variant set. Variants on the Super GGP that were not in the BovineHD variant set were concatenated to the imputed BovineHD set to form a combined scaffold of 31,673 chromosome 5 variants common to all animals. Genotype likelihoods from RTG (GL metrics) were then used to phase the sequence, and subsequently impute the 109,517 sequence variants into the target population. For imputation of the 17 new variants identified through reference assembly refinement (see ‘Sequence mapping and variant calling’ section above), the entire 10 Mbp target region was re-imputed (N = 109,534 variants) to enable direct comparison of association statistics between variants.

### RNA Sequencing

High-depth mammary RNA-seq was conducted using tissue samples from 406 lactating cows, representing three subgroups biopsied at different points through time. Sampling details for 215 of these animals has been described previously^7^, representing two cohorts comprising 27 HFxJ and 188 mostly HF animals. For the remaining 191 animals, five were sampled as part of the above study^7^, but were not previously included in that analysis. The remaining 186 samples were all taken in December 2013, representing mostly HF cows in their third or fourth lactation at mid-lactation (approximately 120 days post calving), producing an average milk yield of 18.4 ± 4.8 (SD) kg/d.

Mammary tissue samples were obtained by needle biopsy and total RNA extracted by NZ Genomics Limited (NZGL; Auckland, New Zealand) using methods described previously^7^,^39^. Sequencing of the 186 animals sampled in December 2013 was performed by the Australian Genome Research Facility (AGRF; Melbourne, Australia) using the Illumina HiSeq 2000 instrument, with libraries prepared using the TruSeq Stranded Total RNA Sample Prep Kit (Illumina) with ribosomal RNA depletion using the Human/Mouse/Rat Ribo-Zero kit (Epicentre/Illumina). All samples were sequenced using a 100 bp paired-end protocol and multiplexed at two samples per lane.

### RNA sequence informatics

#### Read-mapping, expression definitions, and PCA filtering

Sequence data representing all 406 animals was mapped to the UMD 3.1 genome using Tophat2 (version 2.0.12)^40^, locating an average of 88.9 million read-pairs per sample. Cufflinks software (version 2.1.1)^41^ was used to quantify expressed transcripts using the Ensembl genebuild release 72, yielding fragments per kilobase of exon model per million mapped (FPKM) expression values. Genes were considered nominally expressed if they had non-zero FPKM values in at least 75% of samples, and had a mean expression of 0.5 FPKM or greater, yielding 9,348 ‘expressed’ mammary genes. Expression data were also processed using DESeq (v1.14.0)^42^, outputting read counts using the ‘variance stabilising transformation’ (VST) function to render data suitable for linear model analysis. Using these values, individuals were then filtered to remove genome-wide expression outliers based on principle component analysis. Following the guidelines proposed by Ellis *et al.*^43^, individuals greater than three standard deviations from the mean in any of the first six principal components were removed prior to eQTL analyses (375 animals retained).

#### RNA-Seq variant calling

For accuracy assessment of imputed genome sequence data (see ‘Imputation accuracy assessment’ section below), SNP variants were called on the same Tophat2-derived alignments used for expression analysis using SAMtools (v0.1.19), excluding non-primary reads and reads which failed quality filters. Variants were filtered post-calling to retain variants with a) quality scores greater than 100; and b) an average depth per animal of at least five reads. Genotypes for individual animals were set to missing for calls with fewer than 10 reads. This variant set was also filtered to remove SNPs with fewer than 10 alternative alleles called, and fewer than 400 alleles called in total.

### Imputation accuracy assessment

Prior to comparison with imputed genome sequence data, RNA-seq variants were quality-filtered to derive a robust ‘true-positive’ dataset. Starting with the full RNA-seq dataset of 406 cows, there were 3477 RNA-seq variants in the chr5:88945655-98945655 10 Mbp interval of interest. Applying filters for minimum per genotype and per individual call rates of 90%, a MAF threshold of 0.02, and a Hardy-Weinberg equilibrium threshold of *P = *1 × 10^−6^ yielded 994 variants and 386 cows for comparison. For imputed sequence data, a MAF filter of 0.005 was applied in accordance with the criteria used for association analysis. This filter yielded 69,160 variants with a 790 variant intersect with the RNA-seq-derived call-set (Supplementary Table S3). Genotype concordance, minor allele sensitivity, and minor allele discrepancy was then quantified for these SNPs, based on frequency-centric interpretations of the non-reference sensitivity and non-reference discrepancy definitions implemented in the Genome Analysis Toolkit^44^.

### Genetic association analysis

#### Bayesian genome-wide analysis

Genome-wide association analysis of milk fat percentage was performed using GenSel software (Version 4.53R)^45^. This analysis used a population of 42,933 cattle largely overlapping with those described in a previous study^7^, and were a subset of the animals used for imputed sequence association analysis. Animals were typed across a range of SNP-chip platforms (see ‘DNA Extraction and SNP-chip Genotyping’ section above), and imputed using 712,164 SNPs from the Illumina BovineHD BeadChip from a reference population of 796 animals. Pairwise LD-based pruning was performed using Plink software (v1.07^46^) implementing the following parameters: window size = 250, step size = 50, R^2^ > 0.99999. These data were also filtered to exclude SNPs below a minor allele frequency of 0.001, yielding 437,255 SNPs for further analysis. Markers were fitted simultaneously by running a Bayes B model^47^ with a pi value of 0.998. This model was run for 50,000 iterations and included a burn-in of 20,000 iterations, with pi and genetic and residual variance priors first estimated by running Bayes Cpi for 20,000 iterations. R-inverse weights were applied according to the reliability of the phenotype, and covariates for birth year and breed were included in the models. Breed was fitted as the proportions of NZ Holstein-Friesian ancestry, overseas Holstein-Friesian ancestry and Jersey ancestry where the breed proportions were derived from data stored on the New Zealand National Dairy Cattle Database. This database includes all known pedigree and breed sixteenths for the majority of New Zealand diary animals since the 1940s. In addition to considering effects for individual markers, the genome was also partitioned into 1 Mbp windows and the combined effects of SNPs within these intervals were estimated. Supplementary Table S2 summarises the top 50 genome-wide results for these windows.

#### Sequence-based association analyses of milk composition

Associations between SNPs and the milk fat percentage phenotype were quantified by restricted maximum likelihood (REML) using pedigree-based mixed models in ASReml-R^24^. Each SNP was fitted in a separate model, with SNP treated as a quantitative variable based on the number of allele copies. Pedigree relationships were accounted for by fitting a pedigree-based relationship matrix. Covariates were also included for the proportions of NZ Holstein-Friesian ancestry, US Holstein-Friesian ancestry, Jersey ancestry, and heterosis effects. The proportion of phenotypic variance explained by each SNP for each phenotype was calculated using (2p(1-p)a^2^)/t, where p is the frequency of the A allele, a is the estimated allele substitution effect, and t is the total phenotypic variation. Similarly, the proportion of genetic variance explained by each SNP was calculated using (2p(1-p)a^2^)/g, where g is the total genetic variance.

For computational efficiency, association analysis of milk fat percentage was restricted to a 2 Mbp interval centred on the top chromosome 5 tag-SNP identified through Bayesian GWAS (chr5:92945655-94945655). This interval contained 24,283 variants, and additional filters to remove variants with a MAF < 0.005 (N = 8,807), and multiallelic variants (N = 532), yielded 14,944 variants for analysis. Following reference assembly refinement and discovery of new variants within the *MGST1* sequence gap, re-imputation and filtering using the same criteria above yielded 14,985 variants. Analyses for the other milk composition traits (fat yield, protein percent and yield, lactose percent and yield, and milk volume) used a further refined, 50 kbp interval of filtered sequence variants. This interval was centred on the top associated *MGST1* sequence SNP g.93945738C > T (chr5:93920738-93970738), yielding 632 variants for analysis. Significance of tests were assessed using a Bonferroni correction for multiple hypothesis testing (alpha = 0.05). For analysis using the 2 Mbp interval of variants, a threshold of *P* < 3.34 × 10^−6^ was used, for analysis using the 50 kbp interval, a threshold of *P* < 7.91 × 10^−5^ was used.

#### Gene expression analyses

Analysis of gene expression traits was conducted using ASReml-R^24^, using the same pedigree-based mixed models applied for analysis of milk composition traits, with the addition of a fixed effect for biopsy year to account for the differences in cohorts/sequencing facilities used for the three sequencing submissions. For *cis* eQTL mapping in the 2 Mbp milk fat percentage interval, these models used VST-normalised read counts representing the six nominally expressed genes. A total of 14,971 imputed sequence variants were assessed in 375 PCA-filtered individuals (see ‘Read-mapping, expression definitions, and PCA filtering’ section above). This variant set was also restricted to biallelic variants with a MAF > 0.005. Similar models were tested for *trans* eQTL analysis, extending the number of phenotypes tested to all expressed genes (N = 9,348), but restricting analyses to the g.93945738C > T SNP only. For *cis* eQTL analyses, a multiple testing threshold of *P* < 3.31 × 10^−6^ was used (single phenotype, 15,114 variants); for *trans* eQTL analyses, a threshold of *P* < 5.35 × 10^−6^ was used (9,348 phenotypes, single genotype).

#### QTL correlation analysis

Rank correlation analysis of variant *P-*value results lists was conducted to provide insight into whether co-locating QTL possessed the same underlying genetic signal. Use of this method has been described previously^7^, and assumes that pleiotropic QTL will produce similar patterns of association for a given set of variants, as reflected by the relative ranking of the variants’ association statistics. For the current analysis, a 50 kbp window of sequence variants centred on the top milk fat percentage and *MGST1* expression-associated SNP g.93945738C > T (chr5:93920738-93970738; N = 632 variants) was used to quantify Spearman’s rank correlation coefficients. Correlation analyses were performed between milk fat percentage and *MGST1* expression, and the six other milk composition traits (Supplementary Tables S5 and S7).

### CNV analysis

Following observation of read depth anomalies upstream of the *MGST1* gene, we performed read depth-based CNV analysis of the implicated region using CNVnator v0.3^28^. A bin size of 150 bp was selected based on the average read depth and read depth distribution of the sequenced samples. Breakpoints of the ~8 kbp putative CNV were confirmed by PCR (see ‘PCR and targeted sequencing’ section below), with CNVnator then used to call genotypes on all genome-sequence reference animals (N = 556) using a 7 kb interval nested within the deleted segment. Assigned copy numbers were manually visualised revealing a tri-modal distribution strongly suggestive of a biallelic deletion. To assess the accuracy of the CNVnator-derived deletion calls, we examined the haplotype structure of the locus in the 182 genome-sequence reference animals that had also been genotyped using the Illumina BovineHD BeadChip. Comparison of CNVnator-assigned calls and BovineHD genotypes revealed a perfectly predictive 95 kb haplotype (Supplementary Table S11), supporting the integrity of the sequence-based calls. To test the variant for association with *MGST1* expression, this haplotype was used to conduct haplotype-based eQTL analysis in the RNA-seq cohort, using models identical to those used for SNP-based analyses.

### PCR and targeted sequencing

Investigation of sequences representing the MGST1 reference gap and CNV boundaries was performed by PCR and Sanger sequencing. For the reference gap, CACAGAGTTGGACACGACTGA (F1) and CAGTGTTAACCACATTGTTCTTG (R1) primers (Supplementary Fig. S12) were designed to amplify the 364 bp ‘N’ sequence (chr5:93942388-93942751) using Kapa 2G robust enzyme in conjunction with the Kapa GC-rich buffer (Kapa Biosystems). Cycling conditions were: 95 °C for 3 min; and 35 cycles of 95 °C for 30 sec, 59 °C for 30 sec, 72 °C for 30 sec. Amplification of the gap yielded a single band of anticipated size based on *de novo* assembly of short read data (see ‘Locus-specific assembly refinement’ section above), with Sanger sequencing confirming a non-reference gap sequence of 410 bp. For analysis of the CNV junctions, PCR products were designed to span the breakpoints apparent from whole-genome sequence alignments (Supplementary Fig. S12), using the following primers: F2 TCGAAAGGCTGGCACTGACAACGA, R2 GCAGCTGCAGTAGCACATAT, F3 TGGCTGAGTAATACTGATCTGCC, R3 TCCCCACTTTCCCCTTTACT. These reactions used the Kapa 2G HS enzyme in conjunction with the Kapa GC-rich buffer (Kapa Biosystems) and used the following cycling parameters: 95 °C for 3 min; and 35 cycles of 95 °C for 30 sec, 56 °C for 30 sec, 72 °C for 90 sec. Sequencing of the junctions revealed a 8,202 bp deletion abridging two ART2A RTE-BovB repeat fragments. An identical 129 bp consensus sequence within these flanking fragments made the precise breakpoints unresolvable, though occurred somewhere between the chr5:93951990-93952118 and chr5:93960192-93960320 fragments. The presence and/or absence of amplification products for the F2/R2 and F2/R3 junction products was also used to genotype 13 whole genome–sequenced bulls of representative CNVnator-called genotype classes, confirming concordance between PCR and sequence-based calls. All Sanger sequencing was performed using the Applied Biosystems 3130 × L instrument (Life Technologies/Applied Biosystems) at the University of Auckland DNA Sequencing Facility (Auckland, New Zealand).

### Conservation analysis and transcription factor binding site predictions

Gain or loss of putative transcription factor binding sites at the 15 top SNP loci was predicted using MATCH^29^ in conjunction with the TRANSFAC (Release 7.0) database, using the ‘vertebrates’ matrix group with default cut-off to minimise false negatives. The liver-specific binding profile was selected due to being the most lipogenic tissue of the predefined tissue profiles. Predictions were also performed with cut-off settings to minimise the sum of false positive and false negative error rates. Binding sites with core match scores exceeding 0.995 under both conditions, and core motifs overlapping the SNP locus were retained for conservation analysis. Evolutionary conservation was assessed by retrieving orthologous sequences from 100-species alignments from UCSC^48^, and/or local alignment for selected species obtained with ClustalW^49^.

## Additional Information

**How to cite this article**: Littlejohn, M. D. *et al.* Sequence-based Association Analysis Reveals an *MGST1* eQTL with Pleiotropic Effects on Bovine Milk Composition. *Sci. Rep.* **6**, 25376; doi: 10.1038/srep25376 (2016).

## Supplementary Material

Supplementary Information

Supplementary Dataset 1

Supplementary Dataset 2

Supplementary Dataset 3

Supplementary Dataset 4

Supplementary Dataset 5

Supplementary Dataset 6

Supplementary Dataset 7

Supplementary Dataset 8

Supplementary Dataset 9

Supplementary Dataset 10

## Figures and Tables

**Table 1:** Milk fat percentage association statistics for top 17 sequence variants.

Variant Name	Chr5 Pos	REF	ALT	Parameter Est	Parameter-adjusted means			Pheno Var	Geno Var	Corr Top	*P-*value
					Geno_0	Geno_1	Geno_2				
Chr5_93945738	93945738	C	T	−0.1065 ± 0.0029	4.898	4.792	4.685	2.78	4.57	1	1.98E-292
Chr5_93945991	93945991	A	G	−0.1052 ± 0.0029	4.902	4.797	4.692	2.78	4.58	0.93	1.92E-291
Chr5_93948357	93948357	T	C	−0.1052 ± 0.0029	4.900	4.795	4.690	2.76	4.54	0.95	6.16E-289
Chr5_93949810	93949810	A	G	−0.1051 ± 0.0029	4.901	4.796	4.691	2.77	4.55	0.92	1.19E-288
Chr5_93954748	93954748	C	T	−0.1037 ± 0.0029	4.898	4.794	4.691	2.67	4.40	0.92	1.51E-281
Chr5_93954751	93954751	T	G	−0.1037 ± 0.0029	4.898	4.794	4.691	2.66	4.40	0.92	2.10E-281
Chr5_93946027	93946027	A	T	−0.0973 ± 0.0028	4.901	4.804	4.707	2.47	4.08	0.85	5.73E-266
Chr5_93944937	93944937	C	T	−0.1069 ± 0.0031	4.888	4.781	4.675	2.56	4.22	0.83	4.66E-265
BovineHD0500026662	93945655	G	T	−0.1068 ± 0.0031	4.888	4.782	4.675	2.56	4.22	0.83	8.89E-265
Chr5_93948718	93948718	C	G	−0.0969 ± 0.0028	4.900	4.803	4.707	2.44	4.04	0.85	1.84E-263
Chr5_93948646	93948646	G	C	−0.0969 ± 0.0028	4.900	4.803	4.707	2.43	4.03	0.85	3.80E-263
Chr5_93948804	93948804	C	T	−0.0969 ± 0.0028	4.900	4.803	4.706	2.43	4.02	0.84	8.21E-263
Chr5_93947989	93947989	A	T	−0.0969 ± 0.0028	4.900	4.803	4.707	2.43	4.02	0.85	1.05E-262
Chr5_93947761	93947761	T	C	−0.0969 ± 0.0028	4.900	4.803	4.707	2.43	4.02	0.85	1.19E-262
Chr5_93948725	93948725	C	G	−0.0969 ± 0.0028	4.900	4.804	4.707	2.42	4.01	0.85	5.10E-262
Chr5_93946570	93946570	A	G	−0.0969 ± 0.0028	4.899	4.802	4.706	2.43	4.02	0.86	5.52E-262
Chr5_93946548	93946548	C	G	−0.0969 ± 0.0028	4.899	4.803	4.706	2.43	4.01	0.86	1.10E-261

Association statistics for the cluster of 17 top milk fat percentage-associated variants identified through sequence-based analysis. The position and reference and alternate alleles of these SNP variants (UMD3.1) are indicated, with parameter estimates shown with standard errors in units of percentage lipid. The genetic and phenotypic variance explained by each SNP, along with the parameter-adjusted means for each of the three genotype classes is indicated. The linkage disequilibrium R^2^ value for each SNP relative to the top associated g.93945738C > T variant is shown, with the trait association *P-*values indicated in the right-most column.

**Table 2:** *MGST1* locus effects on milk composition phenotypes.

Phenotype	Animal N	Parameter Estimate	Parameter-adjusted means			Pheno Var	Geno Var	*P*-value
			CC	CT	TT			
Fat %	64139	−0.107 ± 0.0029	4.898	4.791	4.684	2.78	4.57	1.98E-292
Fat Yield (g)	64099	−3.135 ± 0.3166	738.7	735.6	732.4	0.22	0.89	4.07E-23
Protein %	64141	−0.031 ± 0.0013	3.817	3.786	3.755	1.10	1.93	1.17E-114
Protein Yield (g)	64105	2.575 ± 0.2392	584.0	586.6	589.1	0.26	1.03	5.20E-27
Lactose %	51120	−0.003 ± 0.0005	5.147	5.144	5.141	0.10	0.23	1.07E-09
Lactose Yield (g)	51330	7.059 ± 0.466	801.0	808.0	815.1	0.64	2.25	1.01E-51
Milk Volume (L)	64105	0.175 ± 0.0091	15.595	15.770	15.945	0.83	2.70	8.13E-83

Association results for the g.93945738C > T variant with milk composition phenotypes are shown, with the number of individuals used for each analysis indicated in the ‘Animal N’ column. Parameter estimates (daily values) are displayed with standard errors using units of grams for yield traits, and litres for milk volume; parameter-adjusted means are given in the same units. The percentage of phenotypic and genetic variance explained by the g.93945738C > T SNP in each model is also indicated, with phenotype association *P-*values shown in the right-most column.
