# Supplementary material for: Sequence-based Association Analysis Reveals an MGST1 eQTL with Pleiotropic Effects on Bovine Milk Composition
Source: Sci Rep. 2016 May 5;6:25376. doi: 10.1038/srep25376 (PMC4857175; doi:10.1038/srep25376)
Supplement: Supplementary Information [file srep25376-s1.pdf]

Supplementary Information from:

Sequence-based Association Analysis Reveals an *MGST1* eQTL with Pleiotropic Effects on Bovine Milk Composition

Mathew D Littlejohn[1,2*], Kathryn Tiplady[1], Tania A Fink[2], Klaus Lehnert[2], Thomas Lopdell[2], Thomas Johnson[1], Christine Couldrey[1], Mike Keehan[1], Richard G Sherlock[1], Chad Harland[1], Andrew Scott[1], Russell G Snell[2], Stephen R Davis[1] & Richard J Spelman[1]

1. Livestock Improvement Corporation, Hamilton, New Zealand
2. School of Biological Sciences, University of Auckland, Auckland, New Zealand

* Corresponding author

Supplementary Legends

Table S1: Chr5 SNP effects. Table showing chromosome 5 SNP effects on milk fat percentage estimated in Bayes B GWAS.

Table S2: Top 50 milk fat percentage QTLs. Table showing top 50 genome-wide QTL windows for milk fat percentage estimated in Bayes B GWAS.

Table S3: Concordance of imputed sequence variants. Table showing imputed sequence concordance values for 790 SNPs in the chr5:88945655-98945655 imputation interval.

Table S4: Milk fat percentage sequence association statistics. Table showing milk fat percentage association results for 14,944 sequence variants in the chr5:92945655-94945655 interval.

Table S5: Milk fat percentage sequence association statistics - adjusted for g.93945738C>T. Table showing milk fat percentage association results for 14,943 sequence variants in the chr5:92945655-94945655 interval, with g.93945738C>T fitted in the models.

Figure S6: Manhattan plot of milk fat percentage associations - adjusted for g.93945738C>T. Manhattan plot of milk fat percentage association results in the chr5:92945655-94945655 interval, with and without g.93945738C>T fitted in the models.

Table S7: Milk composition sequence association statistics. Table showing milk composition association results for 632 sequence variants in the

chr5:92920738-94970738 interval. Traits represented include milk fat percentage and yield, milk protein percentage and yield, milk lactose percentage and yield, and milk volume.

Table S8: eQTL sequence association statistics. Table showing gene expression association results for 14,971 sequence variants in the chr5:92945655-94945655 interval. Genes represented include *MGST1, DERA, COX6B1, STRAP, SNORA23,* and *EPS8.*

Table S9: *Trans* eQTL association statistics. Table showing *trans* eQTL association results for the g.93945738C>T SNP in conjunction with 9,348 mammary expressed genes.

Table S10: *MGST1* eQTL results using a gap-filled, re-imputed variant set. Table showing milk fat percentage and *MGST1* expression association results for 14,985 re-imputed sequence variants in the chr5:92945655-94945655 interval.

Table S11: A CNV-predictive 95kb haplotype. Table showing Illumina BovineHD SNP information (and phase) for the CNV-predictive, 95kb haplotype

Figure S12: CNV and reference gap primer and amplicon design strategy. Primer design details for the *MGST1* intron 1 reference gap, and 5’ deletion variant

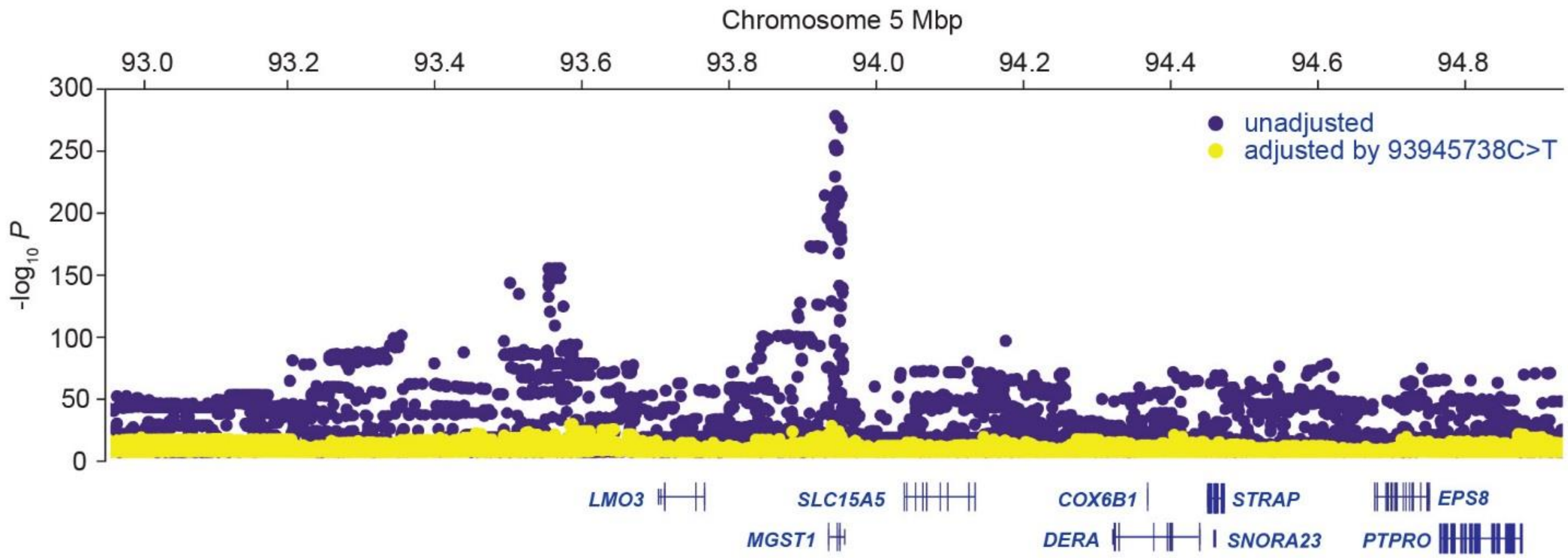
Chromosome 5 Mbp
93.0
93.2
93.4
93.6
93.8
94.0
94.2
94.4
94.6
94.8
300
250
200
150
100
50
0
-log10 P
unadjusted
adjusted by 93945738C>T
LMO3
SLC15A5
COX6B1
STRAP
EPS8
MGST1
DERA
SNORA23
PTPRO

Figure S6

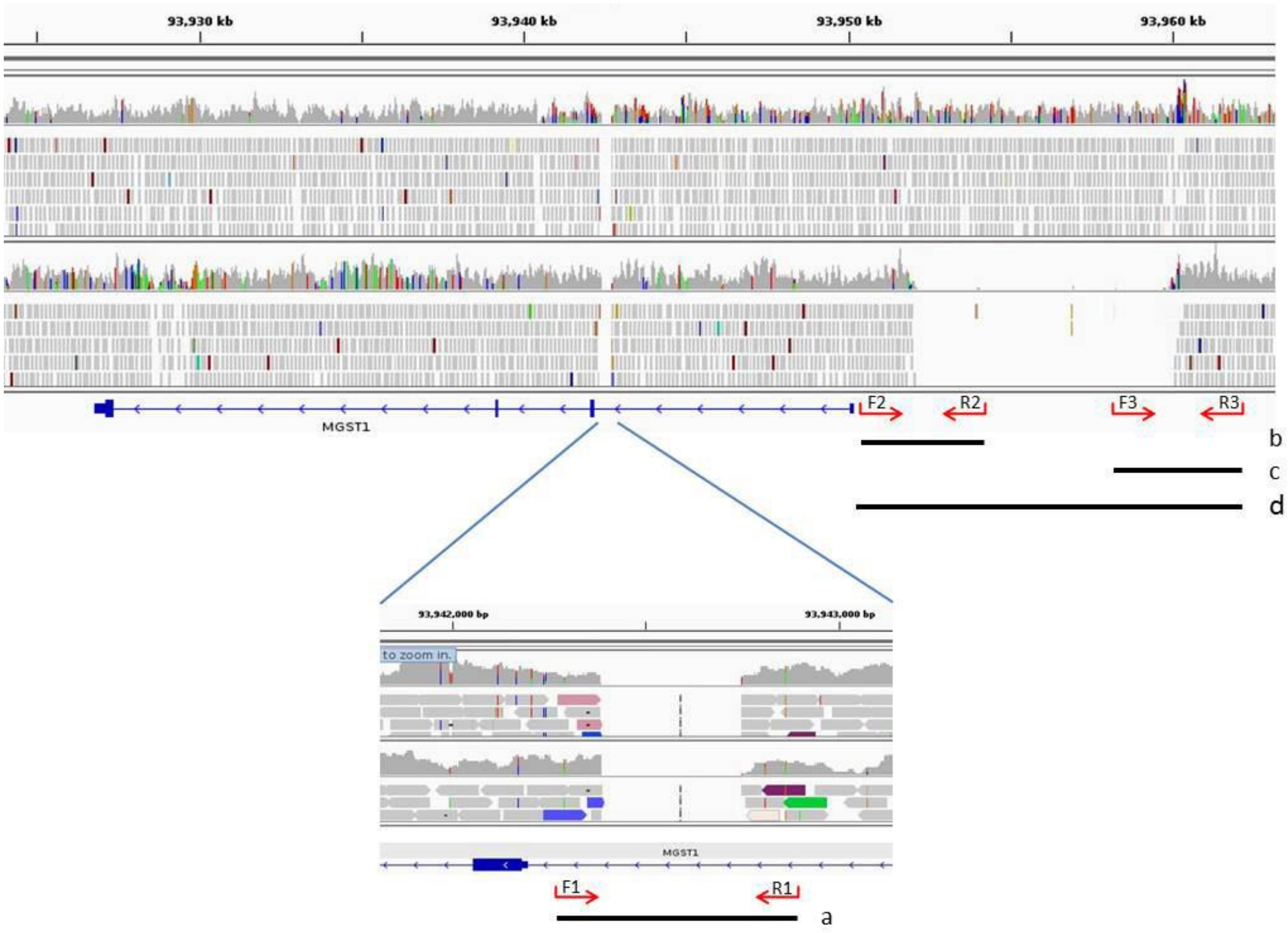

93,930 kb
93,940 kb
93,950 kb
93,960 kb
MGST1
F2
R2
F3
R3
b
c
d
93,942,000 bp
93,943,000 bp
to zoom in.
MGST1
F1
R1
a

Figure S12
